# An Enteric-Coated Polyelectrolyte Nanocomplex Delivers Insulin in Rat Intestinal Instillations When Combined with a Permeation Enhancer

**DOI:** 10.3390/pharmaceutics12030259

**Published:** 2020-03-12

**Authors:** Svenja Sladek, Fiona McCartney, Mena Eskander, David J. Dunne, Maria Jose Santos-Martinez, Federico Benetti, Lidia Tajber, David J. Brayden

**Affiliations:** 1 UCD School of Veterinary Medicine and UCD Conway Institute, University College Dublin, Belfield, Dublin 4, Ireland; svenja.sladek@ucdconnect.ie (S.S.); fiona.mccartney@ucdconnect.ie (F.M.); 2 School of Pharmacy and Pharmaceutical Sciences, Trinity College Dublin, Dublin 2, Ireland; eskandm@tcd.ie (M.E.); djdunne@tcd.ie (D.J.D.); santosmm@tcd.ie (M.J.S.-M.); ltajber@tcd.ie (L.T.); 3 School of Medicine, Trinity College Dublin, Dublin 2, Ireland; 4 ECSIN Laboratory–Ecamricert Srl, Corso Stati Uniti 4, I-35127 Padova, Italy; f.benetti@ecamricert.com

**Keywords:** insulin, hyaluronic acid, chitosan, oral peptide delivery, intestinal permeation enhancers, nanomedicine

## Abstract

The use of nanocarriers is being researched to achieve oral peptide delivery. Insulin-associated anionic polyelectrolyte nanoparticle complexes (PECs) were formed that comprised hyaluronic acid and chitosan in an optimum mass mixing ratio of 5:1 (MR 5), followed by coating with a pH-dependent polymer. Free insulin was separated from PECs by size exclusion chromatography and then measured by HPLC. The association efficiency of insulin in PECs was >95% and the loading was ~83 µg/mg particles. Dynamic light scattering and nanoparticle tracking analysis of PECs revealed low polydispersity, a negative zeta potential range of −40 to −50 mV, and a diameter range of 95–200 nm. Dissolution studies in simulated small intestinal fluid (FaSSIF-V2) revealed that the PECs were colloidally stable. PECs that were coated with Eudragit^®^ L-100 delayed insulin release in FaSSIF-V2 and protected insulin against pancreatin attack more than uncoated PECs. Uncoated anionic PECs interacted weakly with mucin in vitro and were non-cytotoxic to Caco-2 cells. The coated and uncoated PECs, both concentrated further by ultrafiltration, permitted dosing of 50 IU/kg in rat jejunal instillations, but they failed to reduce plasma glucose or deliver insulin to the blood. When ad-mixed with the permeation enhancer (PE), sucrose laurate (100 mM), the physicochemical parameters of coated PECs were relatively unchanged, however blood glucose was reduced by 70%. In conclusion, the use of a PE allowed for the PEC-released bioactive insulin to permeate the jejunum. This has implications for the design of orally delivered particles that can release the payload when formulated with enhancers.

## 1. Introduction

Research on polymeric nanoparticles (NPs) to solve the delivery challenges for oral peptides has increased [1,2]. Particular focus is on NPs prepared with naturally occurring or semi-synthetic polymers [3,4], since many such biomaterials are recognized as safe, with a history of use in humans, and they are used as excipients in oral drug formulations. Slow degradation along with controlled release of peptides in the gastrointestinal tract (GIT) can be achieved with selected polymers [5]. Many of the polymers of interest are polyelectrolytes, which consist of repeated monomers of positive or negative charge. Alginates, carrageenan, chondroitin, and hyaluronic acid (HA) are amongst the anionic polymers, while the cationic ones include chitosan (CS), protamine, and polyarginine-based structures [6,7]. HA and CS have attractive features for oral peptide nanocarrier formulations. HA, a glycosaminoglycan that is found in connective tissue, has a good safety record in humans when used in a range of implanted medical device products [8]. HA has been researched as a component of sustained release formulations and also for oral insulin delivery due to its pH-sensitivity and swelling properties [9]. HA-based NPs can transport payloads into epithelial cells by CD-44 receptor-mediated endocytic uptake [10] and this offers potential for targeting. The muco-adhesive polymer, CS, has an established safety profile and it is of interest for application in oral peptide delivery [11]. It induces epithelial tight junction-openings via the interaction with zonula occludens-1 and occludin [12] and moreover, retains this function in vitro when formulated into NPs [13].

When formulating a nanocarrier for peptide delivery, a mild process is important [14]. The biological activity of peptides is defined by their three-dimensional structure, which is disrupted by high temperature, certain solvents, and extremes of pH [15]. The advantages of polyelectrolyte complexes (PECs) include: the use of aqueous solutions, spontaneous formation of complexes without surfactants or cross-linkers at ambient temperature, high peptide association efficiency, as well as low toxicity and good biocompatibility [6]. Extensive research has been done on the parameters that affect the formation of stable PECs. For example, the combination of a cationic and anionic polymer with similar molecular weight (MW) can result in NPs with smaller particle diameter and tighter size distribution when compared to combining the low and high MW ones [16]. Furthermore, the order of adding the components and speed of addition influences particle diameter, size distribution, and aggregation [17]. Crosslinker-free, non-sedimenting complexes, synthesized from combinations of HA, or chondroitin sulphate with CS or protamine have been extensively characterized [18,19,20]. Mixing ratios, total polymer concentration, and the MW of polymers were the main contributing factors to complex formation. Salmon calcitonin (sCT) was the first peptide that was included in complexes made with HA and CS while using the current process. It had a very high association efficiency, which was dependent on mixing ratios, polymer type, and concentration [21]. Although this PEC construct was ultimately not efficacious for oral delivery of sCT, it proved to have anti-inflammatory features when injected by the articular route in an inflammatory murine model [22].

In devising complexes while using insulin as a model for oral peptide delivery, previous attempts have been made to combine either CS or a CS-derivative with a poly-anion, or insulin itself, via either polyelectrolyte complexation or ionotropic gelation. Matching CS with alginate yielded large nano-complexes that were capable of encapsulating insulin, which were bioactive in diabetic rats following intra-gastric gavage [23]. These initial systems formed the basis of combination PEC-insulin systems, including one made from alginate/dextran sulphate and CS/albumin [24]. Other examples include a pH-sensitive NP that formed by combining CS with γ-poly (glutamic acid) [25]. The oral administration of these NPs in enteric-coated capsules to diabetic rats led to decreased blood glucose levels and a relative bioavailability of up to 20% [26]. Finally, insulin nano-complexes comprising thiolated tri-methylated CS also induced a reduction in blood glucose when orally administered to rats [27]. Even though PEC systems appear to be promising in rodent studies, a disadvantage is the low colloidal stability in bio-relevant media of high ionic strength at a pH that is close to the isoelectric point (pI) of constituents [28]. A reduced charge density of components being induced by counter-ions attaching to the NP surface or by decreased ionization can lead to the rapid release of the peptide and to aggregation [29]. Many such systems have low loading capacities and they may not be possible to scale for manufacture [30].

Here, we synthesized a HA/CS-based insulin-associated anionic PEC, which was optimized with a polymer mass mixing ratio (MR) of 5:1. We report a good association efficiency and low interaction with mucin; however, the colloidal stability in bio-relevant media was still relatively weak. An enteric coating polymer, Eudragit^®^ L-100, was used to improve PEC stability, reduce release in acid buffers, and provide protection against peptidases. Even then, it took the addition of an intestinal permeation enhancer, sucrose laurate [31], to enable insulin delivery in rats following intra-jejunal delivery.

## 2. Materials and Methods

### 2.1. Materials

HA (Streptococcus equi sp.; molecular weight (MW) 15000–18000 kDa) was obtained from Sigma-Aldrich (Arklow, Ireland). Sucrose laurate (SL) was donated by Mitsubishi-Chemical Foods Corporation (Tokyo, Japan). Protasan™ ultrapure chitosan chloride salt 113 (CS) (batch # BP-0806-02) with MW ~100 kDa was purchased from FMC Biopolymer, Norway. Human insulin (Insuman^®^, Sanofi Pharma (Frankfurt, Germany) was provided as a gift. Methacrylic acid: methyl methacrylate copolymer (1:1) (Eudragit^®^ L-100) (EL100) was a gift from Evonik, Germany. The human insulin ELISA was purchased from Mercodia (Uppsala, Sweden). The other chemicals were of analytical grade and purchased from Sigma–Aldrich (Arklow, Ireland), unless stated otherwise.

### 2.2. Synthesis of Insulin-Associated Polyelectrolyte Nanoparticle Complexes (PECs)

A 0.1% (*w*/*v*) HA solution was prepared by dissolving 100 mg sodium hyaluronate in 100 mL deionized water for 12 h at room temperature, followed by 2 h of sonication using a 30 W ultrasonic processor (Sonics VC130PB, Sonics and Materials Inc., Newtown, CT, USA). A 0.1% (*w*/*v*) CS solution was dissolved for 2 h in deionized water adjusted to pH = 3 with 1 M HCl. Insulin (pI = 5.3) was dissolved in the acidified CS solution. 2 mL of the dissolved mixture was then added to 10 mL HA solution in a 25 mL glass beaker while using a rate of ~4 mL/2 s with stirring, which yielded an MR of 1:5 for CS: HA. Other MRs were achieved by adapting the volume of CS solution accordingly. PECs were immediately formed, yielding two theoretical insulin concentrations of 100 µg/mL or 500 µg/mL. The dispersion was left to stir for 10 min allowing for stabilisation. In order to provide a coating around complexes, EL100 (6 mg/mL) was dissolved in ethanol solution (97% ethanol/3% water (*v*/*v*)) and it was added to the dispersion of complexes dropwise at a rate of 4 mL over 8 min in a 1:1 (*v*/*v*) ratio to yield a final concentration of EL100 in the PEC dispersion of 3 mg/mL. The ethanol was removed under vacuum by rota-evaporation using a Büchi Rotavapor R-205. For cytotoxicity studies, PEC dispersions were lyophilised with 3% (*w*/*v*) trehalose as a cytoprotectant [32].

### 2.3. Physicochemical Properties of PECs

The mean hydrodynamic diameter of PECs in dispersions that was based on intensity distribution, the derived count rate (DCR) (kilo counts per second; kcps), and the polydispersity index (PdI) were determined by dynamic light scattering (DLS) at 25 °C while using a 173° scattering angle. The zeta potential (ZP) values were calculated from the mean electrophoretic mobility values that were provided by Laser Doppler Velocimetry (LDV), and by applying the Smoluchowski equation. DLS and LDV measurements were performed using a Zetasizer Nano-ZS ZEN3600 that was fitted with a red laser light beam (Malvern, USA). An 800 µl undiluted aliquot per sample was placed into a clear disposable zeta cell (DTS1070) for size and ZP measurements and equilibrated for 1 min at 25 °C in the chamber. The PECs were measured “as made”, in the native medium without further dilution. PECs were measured in the respective medium in the case of stability or release studies, as described below. The measurements for each sample/batch were taken in triplicate and values were adjusted, depending on the dynamic viscosity of the continuous phase. A low frequency vibration viscometer (SV-10 Vibro Viscometer, A&D Company, Limited) was used to measure the dynamic viscosity. The viscometer was calibrated with deionized water and the samples were equilibrated at 25 °C in a water bath.

Nanoparticle tracking analysis (NTA) measurements were performed while using a NanoSight NS 300 that was equipped with a sample chamber with a red laser (λ = 638 nm) and SCMOS camera. The samples were injected into the sample chamber with sterile syringes (Terumo™ plastic syringe, Egham, UK) until the liquid reached the tip of the nozzle. All of the measurements were performed at 25 °C and analysed with NTA 3.1 software (Malvern Instruments, Worcester, UK). The samples were diluted with MilliQ^®^ water (Millipore Ireland, Cork, Ireland) to suit the optimum concentration range of 105–1010 particles/mL [33]. The data were reported as the mean particle diameter ± SD. Routinely, polystyrene nanoparticles were analysed prior to sample analysis to ensure the accuracy of measurements.

### 2.4. Measurements of Insulin Loading in PECs and HPLC Analysis

The association efficiency (AE), peptide loading (PL), and final loading (FL) of insulin in PECs were determined by separating the insulin that was associated with the particles from free insulin using size exclusion chromatography (SEC). 10 mL of sample were transferred to Zetadex-filled SEC columns (Centripure, MWCO: 5 or 25 kDa; emp biotech, Berlin, Germany) and eluted with Milli-Q^®^ water. Fractions of 1 mL were collected and assayed for insulin (by HPLC) and for nanoparticle content (derived count rate (DCR), kilo counts per second (kcps)). A calibration curve relating the DCR to theoretical particle concentration was established to validate the method. A 0.1% (*w*/*v*) dispersion of NPs was diluted (0.001–0.1% (*w*/*v*)) with water and the DCR of each standard was measured during hydrodynamic diameter measurements. Insulin-associated PECs and unloaded PECs were run separately on SEC columns and their concentration in up to 70 fractions was determined in order to evaluate the efficiency of the removal of free insulin from PECs. Fractions free of PECs scatter light comparable to that of water. After the separation step, the AE was determined using:

AE (%) = (Associated Insulin)/(Total Insulin) × 100(1)

where Associated Insulin is the amount of insulin that is associated to PECs.

PL was calculated as the percentage of insulin in the whole formulation by the following equation:

PL (%) = (Associated Insulin)/(Total Ingredients) × 100(2)

where the Associated Insulin is defined as above, while Total Ingredients is the sum of the weights of all materials in the formulation.

FL was calculated by:

FL (µg/mg) = (Associated Insulin (μg))/(Total Ingredients (mg))(3)

where the Associated Insulin (µg) and Total Ingredients (mg) are defined, as above.

The reverse phase HPLC method for insulin quantification was adapted from Dash et al. [34]. A HPLC system (Waters Scientific, Dublin, Ireland) was equipped with a binary HPLC pump system (model 1525), an auto-sampler (model 717plus), and a dual λ absorbance detector (model 2487). Chromatographic separations were carried out on a Hypersil Gold C18 (5 µm, 250 × 4.6 mm) column (Fisher Scientific, Warrington, UK). 25 µl of either standard insulin solutions (0.5–500 µg/mL) or samples were injected, and isocratic elution was carried out at a flow rate of 1 mL/min. The mobile phase consisted of 50 mM H_2_PO_4_ that was adjusted to pH 2.4 with H_3_PO_4_ (85%) (Riedel-de Haën™, Seelse, Germany) and acetonitrile (>99.9% HPLC grade, Fisher Scientific) (70:30 (*v*/*v*)), and the separation was carried out at room temperature. The UV wavelength that was used for insulin detection was 214 nm. Data were collected and processed while using Breeze™ software, Version 3.30 SPA (Waters Scientific).

### 2.5. Colloidal Stability of PECs

The colloidal stability of PECs was determined by monitoring particle size, PdI and ZP in Simulated Intestinal Fluid (SIF) (15.4 mM NaOH, 50 mM KH_2_PO_4_, pH 6.8) with and without pancreatin (1% (*w*/*v*) [35]. The particles were prepared, as described above, and were mixed with the respective medium (2× concentrated) in a ratio of 1:1 (*v*/*v*). The samples were incubated at 37 °C in a water bath at 100 rpm.

### 2.6. PEC Release Studies in Simulated Intestinal Fluids and Proteolytic Sensitivity

In vitro release of insulin from EL100-coated and uncoated PECs was monitored in the fasted state small intestinal fluid (FaSSIF-V2) [36,37]. 5 mL of insulin-associated PECs were placed in a dialysis bag (MW cut off (MWCO): 1000 kDa; Float-A-Lyzer^®^, Spectrum Labs, San Francisco, USA) and then submerged into a receptor compartment with 45 mL of the respective media containing 0.001% (*w*/*v*) methylcellulose to prevent the adsorption of insulin to the tube. The system was maintained at 37 °C by a water bath and the release studies were performed under sink conditions at a stirring rate of 700 rpm. At selected time points, 1 mL aliquots were collected and replaced with fresh media. HPLC determined the insulin concentration.

Data from release studies were fitted to the first order equation where possible:

W = W_∞_(1 − ⅇ^−kt^) (4)

where W is weight released at time t, W_∞_ is the weight released at infinity, and k is the release rate constant, which was calculated using Origin^®^ version 7.5 software [38].

For proteolysis studies, pancreatin was dissolved in SIF (15.4 mM NaOH, 50 mM KH_2_PO_4_, pH 6.8) at a concentration of 1% (*w*/*v*) and 2 mL aliquots were centrifuged at 14,000 rpm for 30 min. The supernatants were collected and further diluted in SIF (3% (*v*/*v*)). The protein content was measured using a fluorescence-based Qubit™ protein quantitation assay (Life Technologies, Carlsbad, CA, USA). Equal volumes of insulin-associated PECs and supplemented SIF were incubated at 37 °C at 300 rpm for 2 h using a Titramax 1000 orbital shaker to assess the protective effect against proteolytic degradation (Heidolph Instruments, Schwabach, Germany). The aliquots were withdrawn at predetermined time points and added to ice cold 0.1 M HCl to stop enzymatic activity. The samples were centrifuged at 10,000 rpm at 4 °C for 10 min and HPLC determined the concentration of non-metabolised insulin. Insulin solution was used as a positive control.

### 2.7. Cytotoxicity Assays of Uncoated Unloaded PECs: (3-(4,5-Dimethylthiazol-2-yl)-5-(3-carboxymethoxyphenyl)-2-(4-sulfophenyl)-2H-tetrazolium) MTS, ATP, and Neutral Red Uptake Assays

Note that, for these studies, we only focussed on uncoated, unloaded PECs, since the EL100 coating is already in the FDA inactive ingredients guide and the insulin effects on cells are well-known. Caco-2 cell culturing was according to Hubatsch et al. [39]. The cells were obtained from the American Type Culture Collection (Manassas, Virginia). They were grown in Dulbecco’s modified Eagle’s medium (DMEM) with 4500 mg/L glucose, 110 mg/l sodium pyruvate, and sodium bicarbonate and phenol red (D6546), supplemented with 10% (*v*/*v*) heat that was inactivated foetal bovine serum, 2 mM L-glutamine, 100 U/mL penicillin, 100 μg/mL streptomycin, and 1% (*v*/*v*) non-essential amino acids (all from Invitrogen™ Biosciences, Dublin, Ireland). Caco-2 cells were cultured on 96-well plates at a density of 2 × 10^4^ cells/well in tissue culture medium and then incubated for 24 h to allow cell attachment.

The effect of PECs on Caco-2 cell proliferation was determined via MTS assay while using a CellTiter 96^®^ AQueous One Solution Cell Proliferation Assay (Promega, Kilkenny, Ireland) [40]. Cells from passage 52–60 were incubated in culture medium for 2 h or 24 h with increasing concentrations of re-suspended thawed lyophilised uncoated unloaded PECs in deionized water and with Triton™ X-100 (0.5% (*w*/*v*)) as a positive control. The IC_50_ values were calculated by fitting results to the Hill equation (GraphPad Prism^®^ 5, San Diego, CA, USA). Cell viability (%) was expressed relative to the absorbance values that were found for the negative control (cells only exposed to culture medium). Each presented value was normalised against an untreated control and was calculated from at least three separate experiments, each of which included three replicates.

The ATP assay on cells from passage 32 was carried out using an ATPlite Luminescence Assay System (Perkin Elmer, Dublin, Ireland) [41]. The positive control was 1 µM staurosporine. The cells were washed with magnesium- and calcium-free PBS (Lonza, Basel, Switzerland) and mammalian cell lysis solution was added in order to extract and stabilize the ATP after treatment. The plates were shaken for 5 min at 700 rpm. The cell lysates were transferred into a white flat-bottom 96-well plate and standards, PBS (without calcium and magnesium), mammalian cell lysis solution, and substrate solution were added. Luminescence measurements were performed with a Luminometer incorporating Gen5 version software (Agilent Technologies, Cork, Ireland).

The Neutral Red (NR) uptake assay was from Sigma–Aldrich (Italy) [42]. Sodium dodecyl sulphate (SDS) (100 µg/mL in culture media) was used as the positive control. After the treatment with PECs, Caco-2 cells from passage 32 were washed with Dulbecco’s PBS and then incubated with 10% NR solution in culture media for an additional 3 h at 37 °C. The cells were then washed with Dulbecco’s PBS to remove excess dye, before solubilization solution was added to dissolve the incorporated dye. The plate was shaken for 45 min at room temperature and absorbance was read at 540 nm with a Synergy 4 microplate reader. Background absorbance was measured at 690 nm and then subtracted from 540 nm measurement.

### 2.8. Mucoadhesion of Uncoated, Unloaded Anionic and Cationic PECs

Mucoadhesion studies were carried out in accordance with a previous protocol [43], but with minor modifications using a Quartz Crystal Microbalance with Dissipation (QCM-D) system (Q-Sense AB, Gothenburg, Sweden). The rationale to use uncoated PECs was that the EL100 coat should be dissolved from the surface when the PECs interact with mucus in the upper GI regions. The samples were perfused using a peristaltic microflow system (ISM 935; Ismatec SA, Glattbrugg, Switzerland). Gold-coated AT-cut quartz crystals with a fundamental frequency of 5 MHz were used as sensors (QSX301, Particular Sciences, Dublin, Ireland) following the deposition of a layer of porcine mucin (Type III, Sigma) to study the interaction between PECs and mucin at a physiological pH. The sensors were mounted in the flow chamber and a mucin solution (25 mg/L in 30 mM NaCl) was perfused for 15 min at 37 °C and at a perfusion flow rate of 0.01 mL/min until a plateau was reached. The cells were then perfused with 30 mM NaCl to remove any unbound mucin. PECs (0.05% (*w*/*v*)) in 30 mM NaCl pH 5 were then pumped through the system. Mucoadhesion was measured up to 40 min in real time by acquisition Q-Sense software, QSoft401 (Q-Sense AB, Gothenburg, Sweden) through changes in frequency and dissipation. 30 mM NaCl (pH 5) was perfused after the introduction of PECs to confirm that effects seen were due to PECs interacting with mucin. The interaction with the mucin layer was further confirmed using a Zeiss optical microscope, Axiovert 200M, (Cambridge, UK) using a 20× objective. The micrographs were captured using a digital camera and AxioVision software (v 4.7; Carl Zeiss, Rugby, UK).

### 2.9. Rat Jejunal Instillations of Insulin-Associated PECs

Insulin-associated PECs in aqueous dispersion had to be concentrated in order to be able to provide a 50 IU/kg dose of insulin for in vivo studies. A known volume of PECs (uncoated or coated) dispersion was ultra-filtrated using an Amicon Ultra-15 Centrifugal Filter Unit 50 kDa (Millipore, Cork, Ireland) by centrifugation at 4500 rpm for 15 min using an Eppendorf 5810 R (Eppendorf, Hamburg, Germany). Comparison of the volume of the PEC suspension before (C_b_) and after (C_a_) concentration allowed for the calculation of a concentration factor (C_f_):

C_f_ = C_b_⁄C_a_ (5)

The concentration of insulin was calculated from the initial amount that was associated with PECs using the C_f_. PEC diameters were evaluated by DLS before and after concentration by reconstituting to the original volume.

In vivo jejunal instillations in male Wistar rats (300–400 g, Charles River Labs, Durham, UK) were performed with ethical approval from the UCD Animal Research Ethics Committee and under the Licence AE18982/P036 from the Irish Health Products Regulatory Authority. The instillations were performed as previously described with minor modifications [44]. Briefly, following a midline laparotomy, the jejunum was identified and tied off at both ends 5–7 cm apart with a size 4 braided silk suture to create a loop. Solutions of 250–400 µL (based on the concentration of insulin in the concentrated dispersion) were instilled into the lumen while using a 1 mL syringe with 30 G needle. In some studies, an equal volume of sucrose laurate (SL) (100 mM) was instilled prior to the solution of PECs and the length of the loop was increased to accommodate the increased volume. The PECs were assessed by DLS for changes in particle features in the presence of SL. Insulin at a dose of 1 IU/kg was administered by sub-cutaneous (s.c.) administration using a 25 G needle in order to calculate relative bioavailability. Glucose levels were determined from blood obtained from the tail vein over 120 min and measured using a glucometer (Accu-chek Aviva^®^, Roche, Basel, Switzerland). The area above the curve (AAC) of blood glucose reduction was calculated using GraphPad^®^ Prism 5 (San Diego, CA, USA) and plotted against time. The relative pharmacological availability of insulin (%PA) was calculated, as follows [45]:

(6) $$\%\mathrm{PA}=\frac{AAC_{\left(inst.\right)}\times Dose_{\left(s.c.\right)}}{AAC_{\left(s.c.\right)}\times Dose_{\left(inst.\right)}}\times 100$$

where *AAC_(inst.)_* is the area above the blood glucose curve over the instillation period and *AAC_(s.c.)_* is the area above the curve versus time after s.c. injection of 1 IU/kg insulin (0–120 min).

The retro-orbital blood samples were abstracted into 0.5 mL Eppendorf tubes and stored at 2–8 °C prior to centrifugation (5000 rpm, 5 min; Spectrafuge™ 16M, VWR, Lutterworth, UK) and serum collection. Serum was stored at −20 °C until being analysed for insulin by ELISA. Serum data were analysed using PKsolver™ software (China Pharmaceutical University, Jiangsu, China) [46] to estimate the pharmacokinetic characteristics while using non-compartmental analysis. Maximum concentration (C_max_) and the time to reach C_max_ (T_max_) were calculated from the serum concentration profiles. The relative bioavailability (%F) of insulin over the 2 h period was calculated, as follows:

(7) $$\%F=\frac{AUC_{\left(inst.\right)}\times Dose_{\left(s.c.\right)}}{AUC_{\left(s.c.\right)}\times Dose_{\left(inst.\right)}}\times 100$$

where *AUC_(inst.)_* is the area under the concentration curve over the 2 h instillation period and *AUC_(s.c.)_* is the area under the serum concentration versus time after s.c. injection of 1 IU/kg insulin (0–120 min).

Post-mortem, the jejunal loops were fixed in 10% (*w*/*v*) formalin and then embedded in paraffin wax. 5 µm tissue sections were cut on a microtome (Leica Biosystems Nussloch, Germany), mounted on adhesive coated slides, stained with haematoxylin/eosin (H & E), and then examined under light microscopy (Nikon Labphoto; Nikon, Tokyo, Japan).

### 2.10. Statistical Analysis

Statistical analysis was carried out using GraphPad Prism^®^ 5 (San Diego, CA, USA). Release, proteolysis, and the instillation study were analysed using two-way ANOVA with Bonferroni’s post-test. Regarding studies on muco-interaction and cytotoxicity, statistical differences were determined by one-way ANOVA followed by Dunnett’s post-test. The differences were considered to be significant at the *p* < 0.05 level.

## 3. Results

### 3.1. Selection of the Unloaded PEC Prototype

PECs with different surface charges, particle diameters, and charge mixing ratio (CMR) values were obtained across a range of MRs of HA:CS. HA has a maximum of 1 charge, which consists of a dissociated carboxylic group. For CS, the charge density depends on the degree of deacetylation. The degree of deacetylation of the CS batch was 83%, therefore CS has 0.83 charges per subunit. The CMR was calculated as the number of negative charges that are contributed by HA divided by positive charges contributed by CS at the corresponding pH of each PEC dispersion. A CMR > 1 indicated the presence of anionic PECs (MR 2.5–10), while a CMR < 1 indicated cationic PECs (MR 1–1.7). CMR = 1 values lead to charge-neutral, colloidally-unstable particles.

**Figure 1:**
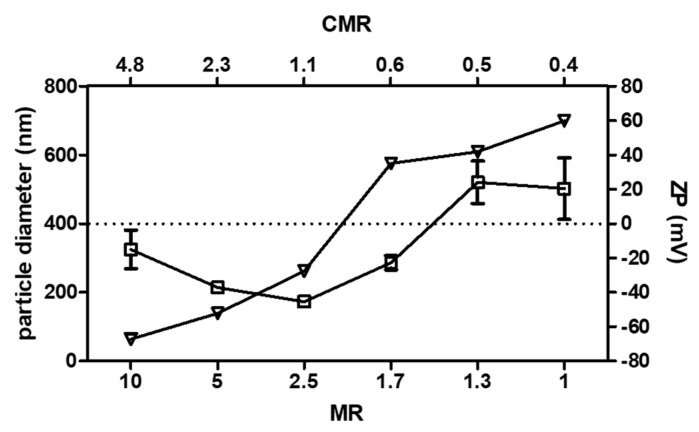
Particle diameter (open rectangles) and zeta potential (ZP) (open triangles) of polyelectrolyte complexes (PECs) in relation to mass mixing ratio (MR) and charge mixing ratio (CMR) of the ratio of hyaluronic acid (HA): chitosan (CS). Data expressed as mean ± SD (n = 3).

Figure 1 shows particle diameters, ZP, and CMR of PECs across a range of MR. Generally, particle diameter ranges between 173 ± 6 nm (MR 2.5) and 521 ± 62 nm (MR 1.3). The particle diameter decreased towards a CMR = 1. The PdI values were between 0.20–0.36 (data not shown), while ZP ranged from −67 mV to +60 mV. A trend towards more positive ZP values was evident with an increase of CS. PECs that were intended for the oral delivery of insulin should have the following beneficial features: a small particle diameter, ZP high enough to provide good colloidal stability through repulsive forces, limited interaction with the mucous layer, and high peptide loading [47]. Therefore, the MR 5 prototype (diameter: 176 ± 29 nm, PdI: 0.17 ± 0.06, and ZP: −45 ± 7 mV (n = 35 batches)) was selected for insulin loading, coating with EL100, and in vivo assessment.

### 3.2. Cytotoxicity Assays in Caco-2 Cells

Uncoated, unloaded MR 5 PECs were lyophilized to yield PEC concentrations that are appropriate for cytotoxicity assays. Prior to assays, they were reconstituted in water: particle diameter, polydispersity index (PdI), and zeta potential (ZP) were unchanged from freshly produced PECs (data not shown). Reconstituted uncoated, unloaded PECs (MR 5) were assayed in three cytotoxicity read-outs while using Caco-2 cells as a human intestinal cell line that represents the human intestine in vitro. The cytotoxicity of PECs up to 10 mg/mL was studied to determine a concentration range where PECs might be used safely for in vivo studies.

**Figure 2:**
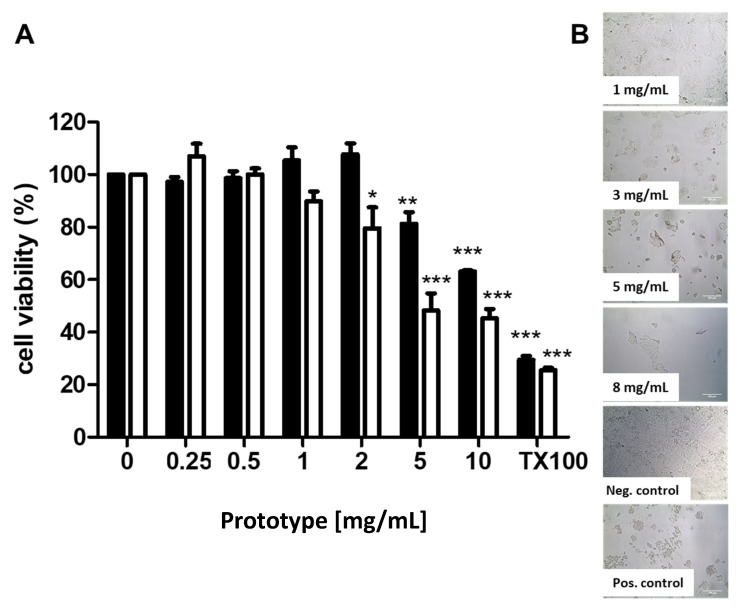
(**A**) MTS assay of PEC prototype MR 5 exposure to Caco-2 cells after incubation for 2 h (black bars) and 24 h (white bars). Data is expressed as mean ± SEM (n = 3–5). 0.01% (*w*/*v*) Triton™ X-100 was the positive control. One-way ANOVA with Dunnett’s post-test (* *p* < 0.05, ** *p* < 0.01 and *** *p* < 0.001 versus untreated control). (**B**) Light microscopy images of Caco-2 cells after 24 h incubation with PEC prototype, MR 5 versus native Caco-2 cells (negative control) and staurosporine (1 µM; positive control). Magnification: ×200. Scale bar = 100 µm.

**Figure A1:**
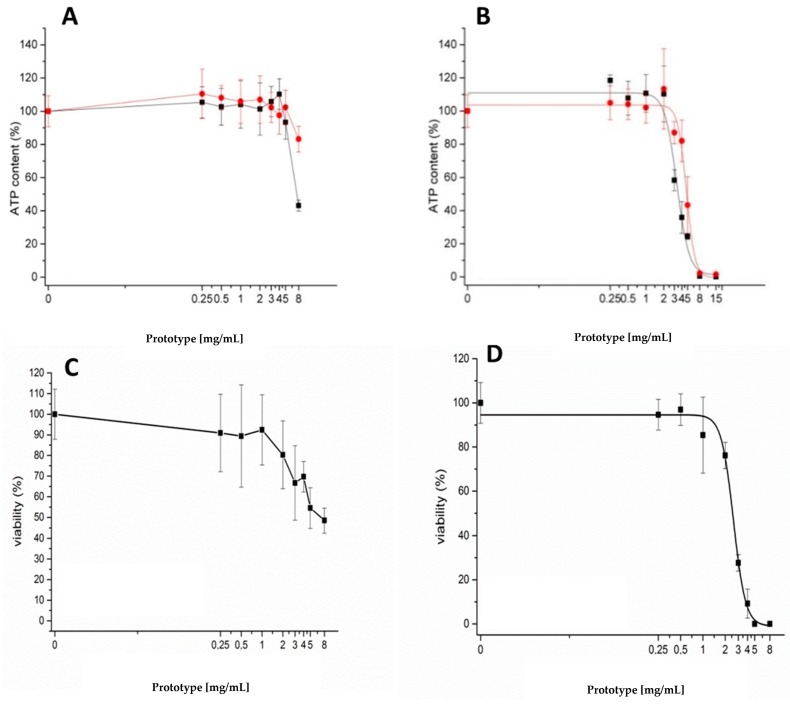
ATP and NR Uptakes assays on Caco-2 cells exposed to different concentrations of PECs. ATP content in unloaded and insulin-loaded PECs (100 µg/mL) was determined after (**A**) 2 h, and (**B**) 24 h. NR Uptake was carried out only with unloaded PECs after (**C**) 2 h, and (**D**) 24 h. Symbols: ■ PEC; ● Insulin-loaded PEC.

After 2 h incubation, PECs induced a reduction in cell proliferation in the MTS assay at 5 mg/mL (Figure 2A), with an IC_50_ of 12.6 mg/mL. After 24 h incubation, the IC_50_ was statistically decreased to 6.2 mg/mL. Optical microscopy confirmed the effects on cell viability after 24 h incubation (Figure 2B). The cells showed altered morphology and cell membrane disruption at concentrations ≥ 3mg/mL over 24 h, which were comparable to the positive control, staurosporine (1 µM). The results were confirmed in Caco-2 cells for PECs using ATP and NR assays (See Appendix Figure A1), where the IC_50_ at 2 h was 7.7 mg/mL (ATP) and 2.3 mg/mL (NR). In the ATP assay, insulin-loaded PECs (100 µg/mL) were also compared to unloaded PECs, and there was no difference (Figure A1). Summarizing the data across three different assays, the PECs were only cytotoxic to monolayers in vitro at very high concentrations of >1mg/mL for 2 h and 24 h exposures. These concentrations were much higher than those that were used in the instillation studies and, in any case, the in vivo epithelium has a higher capacity for repair than cell monolayers [48]. Others have tested CS-coated NPs on Caco-2 cells via MTS assay and concluded that NPs exhibited a cytotoxic effect at ≥1 mg/mL. This value was therefore set as a threshold [49].

### 3.3. Mucoadhesion Studies

**Figure 3:**
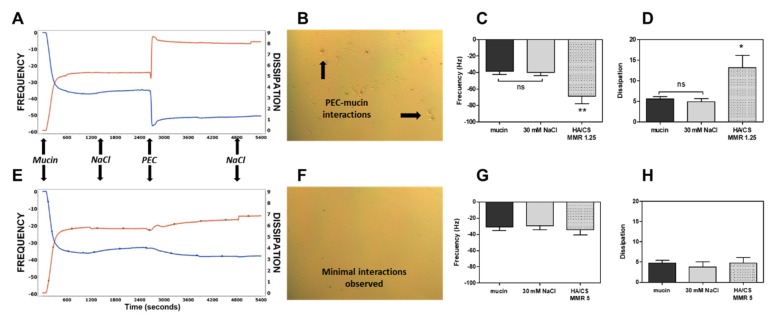
Mucoadhesion of PECs using Quartz Crystal Microbalance with Dissipation (QCM-D). (**A**,**E**) Representative traces from the 5th overtone recorded by the device showing the effects of uncoated, unloaded PECs (MR 1.3) and (MR 5) on frequency (blue line, left axis) and dissipation (red line, right axis). (**B**,**F**) Representative micrographs of the surface of mucin-coated quartz crystals as viewed by phase contrast microscopy (×20). Quantitative analysis of the effects of (**C**,**D**) PECs (MR 1.3) and (**G**,**H**) PECs (MR 5) on frequency and energy dissipation. The data is expressed as mean ± SEM (n = 4). One-way ANOVA with Dunnett’s post-test (ns—not significant, * *p* < 0.05, ** *p* < 0.01 versus control (mucin)).

The effect of uncoated, unloaded PECs (MR 5 and MR 1.3) that were dispersed in 30 mM NaCl solution (pH 5) on mucoadhesion was tested using QCM-D. Figure 3A shows representative traces from the 5th overtone that was recorded by the QCM-D and micrographs of the quartz crystal after the perfusion of PECs, followed by a rinse with NaCl. Frequency and dissipation both showed stable traces after mucin deposition and NaCl perfusion, demonstrating the presence of a stable and reproducible mucin layer. When perfused with cationic PECs (MR 1.3) as a control, a drop in frequency was observed, which suggested the interaction of PECs with the mucin layer (Figure 3A,C; *p* < 0.05). A sharp increase in dissipation was also detected (Figure 3A,D; *p* < 0.05), which was indicative of a change in layer viscoelasticity/thickness during this interaction. A representative micrograph showed the presence of PECs that were attached to the mucin layer (Figure 3B). The same sequence was used to assess anionic PECs (MR 5) and when PECs in NaCl solution were perfused through the system, only a minor change in frequency and dissipation was observed (Figure 3E,G,H), which indicated that there was no mass gain and increase in layer thickness. This effect was further confirmed by optical microscopy that suggested minimal or no interaction between these PECs and the mucin layer (Figure 3F). In summary, cationic PECs (MR 1.3) interact with the mucin layer and the main driving force behind mucoadhesion might be related to the excess amino groups of CS binding to anionic mucin glycoproteins. In contrast, the selected optimum prototype anionic PECs (MR 5) had an excess of HA and were, therefore, less likely to attach to mucin and have higher potential to reach the epithelium once the EL100 coat dissipates.

### 3.4. Insulin Loading of Uncoated and Coated PECs

**Figure A2:**
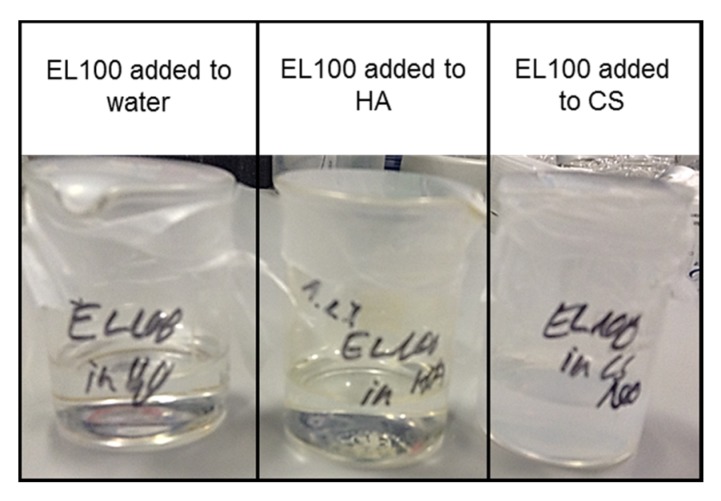
Photographs of samples containing EL100 added to either water, HA, or CS solution in 25 mL glass beakers. Note the clear solutions in water and HA, and the opalescence in CS.

EL100 solution was added dropwise to individual solutions of HA and CS in the same ratios as used in PECs and the resulting dispersions were visually assessed in order to examine the coating process. The addition of EL100 to water and aqueous HA (0.83 mg/mL) resulted in a clear solution, whereas the addition to aqueous CS (0.17 mg/mL) caused opalescence (Figure A2).

Table 1 shows the characteristics of uncoated and EL100-coated PECs (MR 5) theoretically entrapping 100 and 500 µg/mL insulin. The insulin concentrations in acidified CS solution were 0.6 mg/mL (100 µg/mL loading) and 3.0 mg/mL (500 µg/mL loading) in order to achieve these target concentrations. Loaded, coated PECs had a mean diameter of <200 nm (154 nm diameter for 100 µg/mL loading, 116 nm for 500 µg/mL loading) and a homogenous size distribution. The decrease in particle diameter as compared to uncoated prototypes might be due to a second population forming or to shrinkage of PECs after the addition of EL100. The AE for insulin in coated PECs was >95% higher than uncoated PECs (>80%). The PL and FL of coated PECs were less than that of uncoated particles due to additional material present. The PL and FL values were higher for PECs using 500 µg/mL than for 100 µg/mL l insulin starting concentrations, so, therefore, the former concentration was used for uncoated and coated PECs in in vivo studies.

**Figure 4:**
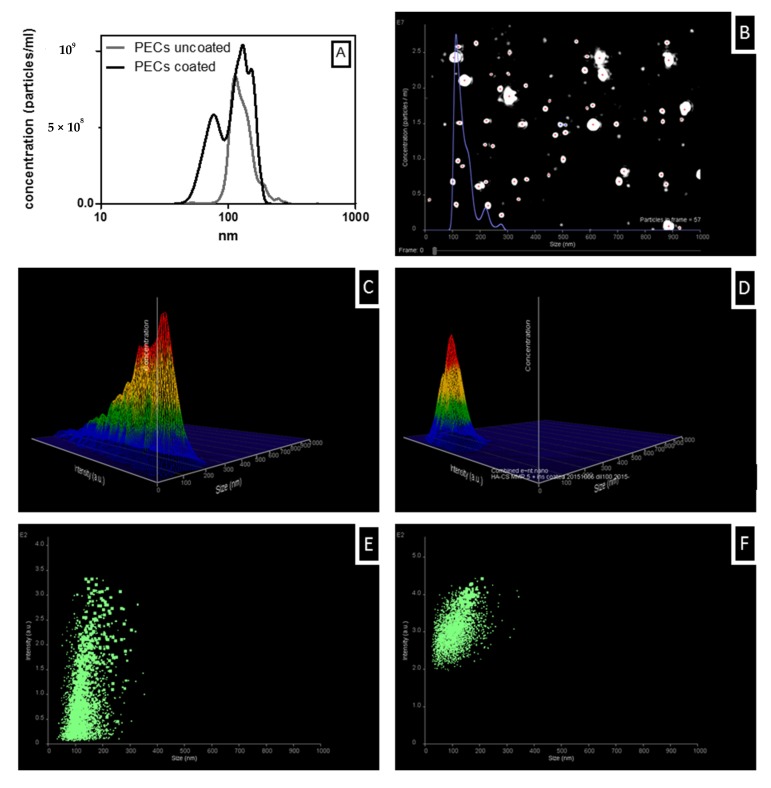
Nanoparticle tracking analysis (NTA) analysis of PECs that were loaded with 100 µg/mL insulin. (**A**) number-based size distributions of uncoated (grey) and coated (black) sample, (**B**) representative video frame of uncoated sample, representative 3D graph (particle diameter vs. intensity vs. concentration of (**C**) uncoated and (**D**) coated PECs, scattergram of intensity (A.U.) vs. particle diameter of (**E**) uncoated and (**F**) coated PECs. Data of number-based size distribution is expressed as mean (n = 2 for both samples, three measurements each).

NTA was also used to confirm size distributions of uncoated and coated PECs that were loaded with 100 µg/mL insulin. The average particle diameter that was measured by NTA across two samples in triplicate was 138 nm for uncoated and 123 nm for coated PECs. When plotting particle diameter against particle concentration, the peak pattern of the uncoated and coated PECs showed differences in the size distribution and concentration (Figure 4A). While uncoated PECs had a unimodal population, the coated PECs displayed a bimodal distribution. The initial peak was ~78 nm, followed by a second peak of ~131 nm, the same particle diameter as uncoated PECs. This might be due to a second population of particles forming that comprises un-complexed CS and/or insulin. Figure 4B shows a typical NTA video frame that is obtained for the uncoated PECs. Three-dimensional (3D) plot and scattergram of uncoated PECs revealed a broad intensity spectrum with multiple peaks and the highest particle concentration at lower intensity values (Figure 4C,E). For coated PECs, the intensity shifted towards higher values and a much narrower range with only two peaks seen (Figure 4D,F).

The difference in particle diameter that was measured by DLS and NTA was likely due to a combination of high dilution for NTA and the different sizing principles of the techniques. Still, overall, the DLS and NTA data confirm that the insulin-loaded coated PECs are in the 100–200 nm range. Finally, the high insulin dose that is required for in vivo studies necessitated an additional centrifugation-ultrafiltration step: a 3–4 fold increase in concentration was typically achieved across PEC batches using this process. The diameters of coated and uncoated insulin-loaded uncoated PEC increased by up to 100 nm and 20 nm, respectively, following this concentration step, but PdI and ZP were unchanged (Table 2).

### 3.5. Colloidal Stability of Uncoated and Coated PECs

**Figure 5:**
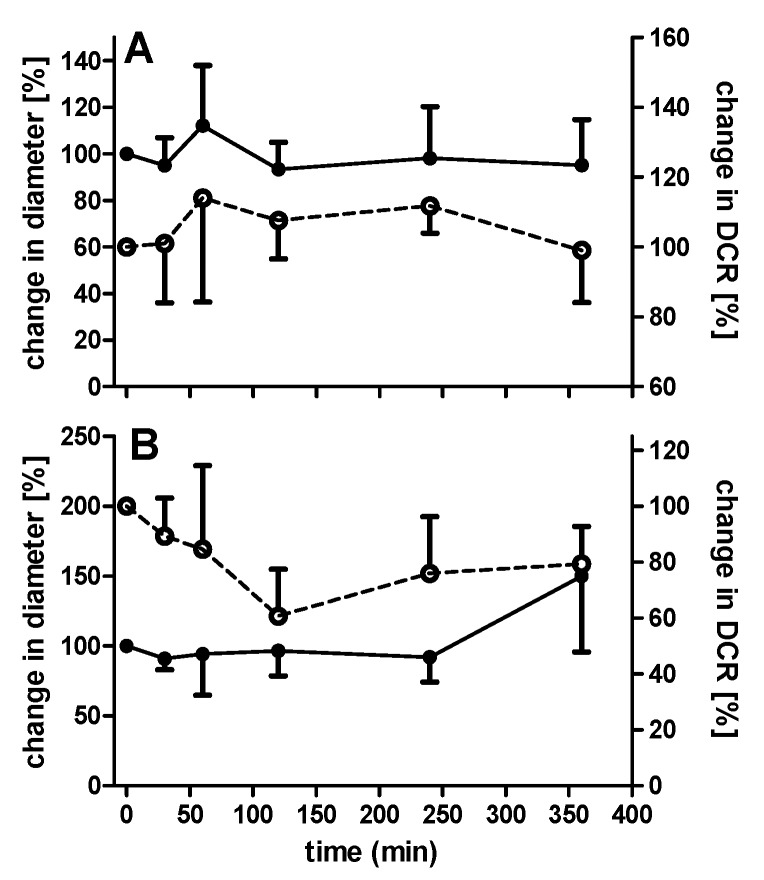
Stability of EL100-coated PECs (100 µg insulin concentration) in (**A**) Simulated Intestinal Fluid (SIF) and (**B**) SIF supplemented with pancreatin. Readout was the % change in particle diameter (solid line) and derived count rate (DCR) as measured by DLS (dashed line). Data expressed as mean ± SD (n = 3–5).

The structural integrity of PECs will need to be maintained until arrival in the small intestine. The behaviour of PECs was tested in SIF (pH 6.8), as lyophilized PECs can potentially be loaded in enteric-coated capsules. The addition of uncoated PECs with a theoretical loading of 100 µg/mL insulin to SIF resulted in an immediate colloidal instability, which was characterized by large increases in diameter and a PdI value of up to 1.0 (data not shown). In contrast, EL100-coated, insulin-loaded PECs were stable in SIF, as indicated by the measurement of diameter and DCR (Figure 5A). The DCR decreased slightly between 4–6 h, which suggested either dissolution or sedimentation.

Subsequently, the stability of the coated PECs in SIF that was supplemented with 1% (*w*/*v*) pancreatin was also monitored and the overall pattern similarly did not indicate particle disruption (Figure 5B). The decrease in DCR over the initial 120 min might have been due to the sedimentation of pancreatin aggregates, as there was no change in diameter. However, over 6 h, an increase in diameter and DCR was observed, which was perhaps due to pancreatic enzymes attaching to the PEC surface. DLS data that were obtained from studies in supplemented SIF need to be interpreted with caution because intensity distribution plots suggested possible interference by pancreatin, due to the presence of particle diameters in the same range as coated PECs. However, when converted to actual number distribution, most pancreatin aggregates were smaller than coated PECs and, in addition, there was a second peak (pancreatin) present for PECs in SIF-supplemented with pancreatin, so it was concluded that the Z-average values that are calculated are indeed predominantly PECs.

### 3.6. PEC Release of Insulin: Studies in FaSSIF-V2

**Figure 6:**
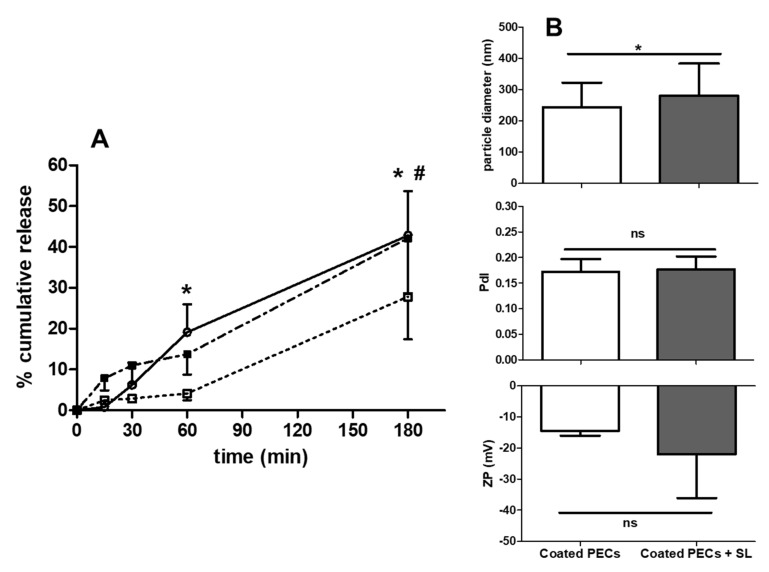
(**A**) Cumulative release of insulin from uncoated PEC (open circle), EL100-coated PECs (open square) and coated PECs + SL (100 mM) (closed square), all loaded with 500 µg/mL insulin, in FaSSIF-V2 at 37 °C. Two-way ANOVA with Bonferroni’s post-test (* *p* < 0.05 uncoated vs. coated PEC, ^#^ *p* < 0.05 coated PECs vs. coated PECs + SL). (**B**) Particle diameter, PdI, ZP of coated PECs loaded with 500 µg insulin with and without the addition of SL in FaSSIF-V2. Data expressed as mean ± SD (n = 3–4). Paired Student’s *t*-test (NS, not significant; * *p* < 0.05 for coated PECs + SL versus coated PECs). Data expressed as mean ± SD (n = 3).

Lyophilized PECs can potentially be filled into enteric-coated capsules, as stated earlier. Therefore, the release profiles of insulin-associated uncoated and coated PECs (MR 5) at a theoretical loading of 500 µg/mL were evaluated in FaSSIF-V2 (Figure 6A).

**Figure A3:**
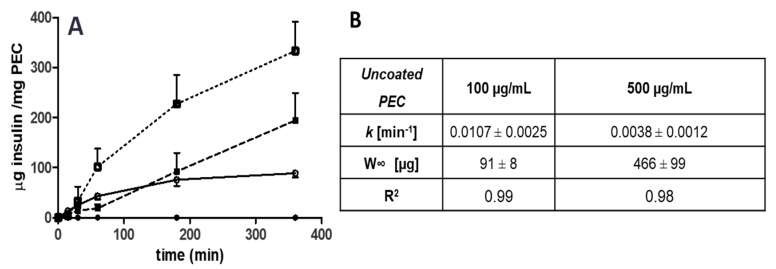
(**A**) Insulin released from uncoated and coated PECs loaded with insulin FaSSIF-V2 over 6 h. Groups: uncoated PEC, 100 µg/mL (o); uncoated PEC, 500 µg/mL (☐); coated PEC, 100 µg/mL (●); coated PEC, 500 µg/mL (■). Data expressed in mean ± SD (n = 3–4). (**B**) Model parameter estimates and correlation coefficient for insulin release data fitted to a first order model, where W∞ is the amount of insulin released at infinity and k is the release rate constant. (n = 3–4).

The uncoated PECs gradually released 43 ± 11% insulin over 180 min, whereas coated PEC released it over two phases: ~4% within 60 min, and 28 ± 10% by 180 min. The release from coated PECs was also assessed when SL (100 mM) was dissolved in the dispersion. The addition of SL led to a similar biphasic release pattern as for the coated PECs alone, but with up to 40 ± 15% released at 180 min, statistically higher than in its absence. The coated PECs were only affected by SL in respect of a slight increase in particle diameter (Figure 6B). These data suggest that SL caused the solubilisation of coated PECs. Overall, the uncoated PECs exhibited a gradual release profile that could be fitted to a first-order release model (R^2^ values = 0.98), however this model could not be applied to the release data for coated PECs, as release was biphasic (Figure A3).

**Figure A4:**
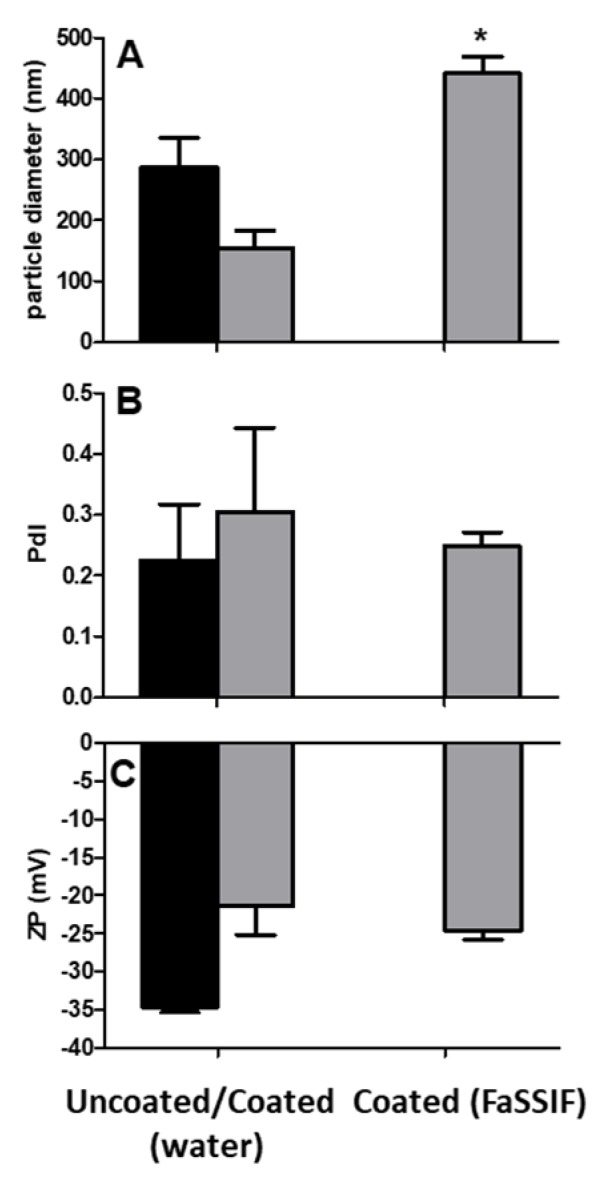
Particle diameter (**A**), PdI, (**B**) and (**C**) ZP of PECs loaded with 100 µg/mL insulin in water at t = 0 and after 6 h incubation in FaSSIF-V2 at 37 °C. ■ Uncoated; ■ Coated. The uncoated PECs gave PdI values ~1 and aggregated in FaSSIF after 6 h. Mean ± SD (n = 3–7). * *p* < 0.05, paired *t*-test. Data was similar for PECs loaded with 500 µg/mL insulin (data not shown).

Finally, uncoated PECs aggregated during incubation, as reflected by an increased PdI (~1.0). In contrast, the diameter of coated loaded PECs increased from ~170 nm to ~400 nm after 6 h incubation, while PdI and ZP were unchanged (Figure A4). Similar to colloidal stability in SIF, the integrity of coated, but not uncoated, PECs was maintained.

### 3.7. Proteolytic Sensitivity

**Figure 7:**
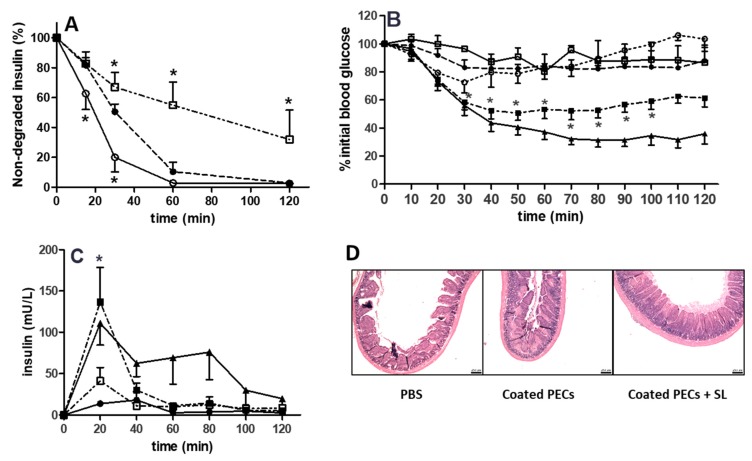
(**A**) Degradation profile of insulin solution as compared to insulin entrapped in coated and uncoated PECs and incubated in pancreatin-supplemented SIF. Prototypes were loaded with 500 µg/mL of insulin. Insulin solution (●), Insulin-loaded uncoated PECs (o), Insulin-loaded coated PECs (□), Data expressed as mean ± SD (n = 3–4). (**B**) Plasma glucose levels following jejunal instillation of insulin (50 IU/kg). Symbols as in A, but with an additional group, insulin-loaded coated PECs co-administered with 100 mM SL (■). S.C. insulin (1 IU/kg) was used as control (▲). The blood glucose levels were standardised to t = 0 min (100%). (**C**) Insulin serum levels following instillations. Symbols as in B, except uncoated PECs were not tested. (**D**) Haemotoxylin and eosin staining of jejunal loops following 120 min instillations. Bars = 250 µm. Two-way ANOVA followed by Bonferroni’s post-test. Mean ± SEM (n = 4–6). * *p* < 0.05, vs. insulin solution in A, B, and C.

Figure 7A shows the degradation of free insulin when compared to insulin-loaded coated and uncoated PECs (MR 5) in the presence of pancreatin-supplemented SIF. The protein content in the supplemented SIF was 38.7 ± 3.7 µg/mL. Over 50% of free insulin solution was degraded by 30 min, and >90% within 1 h. Insulin that is associated with uncoated PECs exhibited even more rapid degradation than free insulin, with 80% being degraded by 30 min. However, coated PECs conferred some protection: by comparison with the profiles of free insulin and insulin-associated with uncoated PECs, 45% of insulin in coated PECs was degraded at 60 min, with 68% at 120 min.

### 3.8. Rat Intra-Jejunal Instillations of Insulin-Loaded PECs

Instillations were carried out while using both uncoated and EL100-coated insulin-loaded PECs using an insulin dose of 50 IU/kg. Neither they, PBS, nor insulin solution significantly reduced the plasma glucose levels, in contrast to the 70% reduction seen with s.c. delivery of 1 IU/kg insulin (Figure 7B). In contrast, when 100 mM SL was instilled with insulin-loaded coated PECs, a reduction in glucose levels was conferred, which was significantly different to the insulin solution at time points beyond 40 min. The %PA revealed a rank order of insulin-loaded coated PECs + SL (1.8%) > insulin-loaded coated PECs (0.7%) and insulin-loaded uncoated PECs + SL (0.7%) > insulin solution (0.4%). When examining serum levels of insulin, the insulin-loaded coated PECs + SL produced the highest levels, although the values were modest (F = 1.2%) (Figure 7C, Table 3). Finally, the histology of jejunal loops was examined following instillations of insulin-loaded coated PECs and insulin-loaded coated PECs + SL (Figure 7D). Neither were damaging to the mucosa and they were similar to the PBS control.

## 4. Discussion

Polyelectrolyte complexation that is based on electrostatic interactions of polymers is a simple scalable process that occurs in an aqueous environment where the formation of nanoparticles is instantaneous. The particle diameter and ZP can be manipulated to suit injected or non-injected formats.

The synthesis of similar PECs using CS and HA, but with sCT, also led to a high AE [21,22,50]. In contrast to sCT, in the current study, insulin was not pre-complexed with HA, but it was initially dissolved in acidified CS solution. The complexation of polyanions and polycations is mainly governed by ionic binding sites, polymer chain rigidity, and the pH and ionic strength of the surrounding media [16]. Other studies using infrared spectroscopy confirmed electrostatic interactions between anionic carrageenan and cationic protamine as the main driving force for complexation [51]. Generally, acceptable PEC characteristics were achieved while using dilute polymer solutions in concentrations of ~0.1% (*w*/*v*) [16,17,32]. The use of depolymerized HA in the current study yielded physically stable PEC dispersions with hydrodynamic diameters of 100–600 nm and PdI values < 0.3. By varying the MR, the particle diameter and ZP values were manipulated in order to yield optimized PECs with an anionic surface that was charge strong enough to prevent instant aggregation and sedimentation in aqueous solution. Previously, HA/CS-based PECs using polymers of similar MW and containing the crosslinker, sodium tripolyphosphate were prepared with MRs from 1:2–2:1 [52]. Others have prepared HA/CS PECs with polymers of higher MW and resulting in ZP values that were between +27 and −21 mV [53]. The particles in [52,53] consisted of a dense, charge-neutralised core surrounded by a less dense corona, mainly containing the polymer and charge in excess. The PECs should ultimately have a charge-neutral core containing both polymers. Sarmento et al. also observed a similar morphology, who found a relatively dense core of dextran sulphate and CS, with excess dextran sulphate also depositing on the surface [54].

We assessed the potential for cytotoxicity of the PECs (MR 5) using a selection of end-point assays in Caco-2 cells: MTS, NR uptake, and ATP depletion. Most of the studies were with unloaded PEC, but some studies were carried out with insulin loaded PECs while using the ATP assay. The most relevant data was for 120 min exposures, as intestinal epithelia will only be exposed for a short time in vivo. The toxicity in vivo is expected to be even lower due to the protective mucus layer in the GIT. Furthermore, the concentration of the PEC prototype showing a reduction in cell viability in vitro after 2 h incubation (5 mg/mL, 20% reduction in viability) was considerably higher than the concentration of the prototype that was used in subsequent in vivo studies (~4 mg/mL). Across these assays, the IC_50_ values were in excess of 1 mg/mL at both 2 and 24 h, thus confirming the innocuous nature of these biomaterials. The cytotoxicity data on PECs were of the same order as that of another prototype that was recently published from TRANS-INT using similar assays that were carried out in the same reference laboratory as for this paper [55].

At acidic pH, insulin has a cationic charge due to deprotonation of amine functional groups of histidine, lysine, and arginine, and is therefore attracted to the anionic carboxylic groups of HA. Almost 100% of CS will be positively charged in an acidic solution since the pKa of CS relating to the amino groups is 6.5 [56]. The pKa of the carboxylic groups in HA is estimated to be 2.9 [57]. However, most of the carboxylic groups of HA in solution would not be ionized in acidic buffer and the interactions with cations would be weak. Consequently, an adjustment to pH = 3 was required in order to maximize ionisation to promote electrostatic attraction between HA and CS. Particle diameter and PdI were affected by acidifying the CS solution, perhaps because CS changes to a fully extended form at this pH, leading to higher chain rigidity when compared to the conformation seen at pH values that were closer to its pKa [58]. Similar changes in particle diameter and ZP were seen in alginate/CS PECs prepared across a pH range of 4.2–5.2 [59], and also in PECs that formed between dextran sulphate and CS [54].

A crucial step was the separation of free insulin from insulin associated with PECs using a non-destructive method. The incorporation of insulin in PECs is based on electrostatic interactions and the loading capacity is influenced by the pI/pKa of the components involved, similar to interaction between polymers. One example studying PECs with excess CS displays an increase in particle diameter due to repulsion and a decrease in the negative ZP with increasing sCT concentration [19]. In the current study, an anionic PEC prototype (MR 5) with an excess in HA for loading studies was chosen. The physicochemical characteristics of PECs were not changed by the complexation of increasing concentrations of insulin, which mainly is consistent with its location in the core and not on the surface. Furthermore, high AE and PL values were achieved.

The PEC diameter is important for ensuring colloidal stability in the small intestinal lumen. However, with decreasing particle diameter, the surface area and total surface energy increase, which makes such nanoparticles thermodynamically unstable, thus leading to Ostwald ripening [60]. The ZP further influences colloidal stability and interaction of NPs with tissue. The plasma membrane of enterocytes contains anionic proteoglycans, thus a positive ZP can, in theory, enhance interaction with PECs, but they may get trapped by anionic glycoproteins in mucus before reaching the epithelium [61]. Nonetheless, a high ZP value is needed to colloidally-stabilize PECs via electrostatic repulsion [62]. In this context, while the anionic PECs (MR 5) were not attracted to mucin and may reach the epithelium, the electrostatic repulsion from anionic glycoproteins in the plasma membrane are not favourable for the epithelial uptake of these PECs.

The stabilization of PECs can be disrupted by changes in pH, ionic strength, charge density, and MR [16,17]. Physical instability in bio-relevant media is one of the major disadvantages of colloidal systems and it can result in secondary aggregation or dissolution. As PECs are held together mainly by electrostatic interactions and stabilized by ionized groups on the particle surface, a small change in the ionic strength or the pH of the surrounding media can change the charge density and interfere with PEC integrity. Furthermore, weak poly-acids, including HA, form complexes with weaker bonds when compared to complexes containing polyelectrolytes with strong acid groups, such as chondroitin sulphate [20]. In SIF, immediate aggregation was accompanied by a charge inversion, whih was most likely induced by phosphates and other anionic electrolytes. Similarly, immediate aggregation was seen when HA/CS NPs crosslinked with tri-polyphosphate (TPP) were introduced into simulated lachrymal fluid [32].

Colloidal stability of HA/CS PECs was improved by coating PECs with EL100. Other common approaches that are found in the literature to improve colloidal stability include CS quarternization [63], ionic crosslinking with TPP [64], covalent crosslinking [65], or coating with poloxamers [66] or PEG [67]. A combination of different approaches might be required, such as “tandem crosslinking” with TPP (ionic) and glutaraldehyde (covalent) [68], or by the quarternization of CS and subsequent disulphide crosslinking between thiolated trimethylated CS and HA [69]. The EL100 coating resulted in a system with improved colloidal stability in bio-relevant buffers. DNA-entrapping CS-based PECs were also successfully coated with EL100 via electrostatic interactions [70]. EL100 is designed to dissolve at the pH ranges of the rat jejunum based on its pH-dependent solubility. Furthermore, complexes comprising thiolated EL100 showed promising insulin loading and low cytotoxicity in the nm size range [71]. Here, after the coating of PECs with EL100, particles decreased in diameter by ~30%. Others have interpreted this as either condensing effect [72], or shrinkage induced by a decrease in electrical repulsion due to the coating [73].

The structural characteristics of the polymer matrix, as well as the interaction of the matrix with the incorporated drug, as well as the solubility and stability of the drug, can influence insulin release kinetics [74]. In the present work, uncoated PECs showed a gradual release of insulin, while the release in coated PECs was biphasic. EL100 in combination with low MW CS is known to swell up to 100% of its size at pH 6.8 within 30 min [75] and, therefore, insulin should be released in the first phase. Subsequently, insulin in the core might diffuse through the damaged coat. Calcetti et al. observed a similar biphasic release pattern, when formulating PEGylated insulin into tablets with thiolated poly(acrylic acid) [76]. Overall, the findings suggest release that is based on the disintegration of PECs and subsequent release of insulin. For this technology, it seems a more likely scenario than particle uptake by the epithelium.

PECs have to prevent the enzymatic degradation by serine proteases in the small intestine in order to enhance oral bioavailability of peptides. The protection of insulin from degradation by pancreatic enzymes was tested in SIF that was supplemented with pancreatin. When associated with uncoated PECs, insulin surprisingly degraded quicker than free insulin. This was also noted when insulin was complexed with amphiphilic polymer, polyallylamine [77]. The phenomenon could be a result of insulin restructuring in the core and becoming prone to hydrolysis by α-chymotrypsin. EL100-coated PECs partially inhibited insulin from degradation by pancreatin, so the coating seems to perform this role in addition to enabling the pH-dependent release in intestinal regions. EL100 might inactivate trypsin, as the latter seems to bind poly(acrylic acid) [78], while chymotrypsin can bind carboxylate units, leading to enzyme inhibition [79].

Even though the coated PECs protected entrapped insulin, there were minimal effects on the plasma glucose levels and insulin bioavailability following rat jejunal instillations. This in vivo technique is a best-case scenario screening assay that must be passed before moving to gavage studies. Nonetheless, the PECs likely released insulin too slowly and inefficiently in the jejunum and they were relatively ineffective in the absence of the enhancer, SL. The instillation of coated prototypes with SL resulted in hypoglycaemia that was accompanied by increased insulin serum levels. It seems that the SL both assists the solubilisation of the PECs and acts directly on the plasma membrane to increase the permeability of insulin [31]. Therefore the rat study confirmed, firstly, that the released insulin was bioactive, secondly, that the coated PECs had sufficient insulin loading to yield a response, and thirdly, that the released insulin had the capacity to cross the epithelium if it was assisted by SL. Arising from this, the primary role of PECs was to protect insulin and assist its passage through mucus to the epithelial wall. While it seems intuitive that nm particles should be endocytosed by the epithelium to a greater extent than their micron sized equivalents, evidence that epithelial PEC uptake occurs in vivo in rat jejunal instillations to any extent was scant: there was no evidence that fluorescein isothiocyanate (FITC)-labelled PECs that were made from HA/CS were taken up to any extent by the rat jejunal epithelium following instillations; some were located in overlying mucus [80]. Consequently, there was no rationale to investigate the particle uptake studies in Caco-2 cells or carry out biodistribution studies of PECs in vivo.

Further formulation development for establishing an optimized release rate needs to be conducted. Additionally, the entrapment of an appropriate permeation enhancer in the PEC formulation that co-releases in high concentration with insulin is suggested. Finally, the concentration of PECs for in vivo study needs further optimization to yield dispersions without aggregates. Heterogeneous PEC dispersions might have resulted in the high intra-batch variabilities that were observed in hypoglycaemia and variable serum insulin levels. Lyophilized insulin-loaded, coated PECs should be administered with SL in coated capsules by oral gavage in a large animal to further develop the prototype [81].

## 5. Conclusions

PECs containing HA and CS with average diameters of 160–500 nm were adapted to maximize the encapsulation of insulin. With an MR of HA: CS of 5:1, the diameters were further narrowed to the 200 nm range with ZPs of −50mV. The anionic PECs showed no aggregation or phase separation. The subsequent coating of insulin-loaded PECs with EL100 increased colloidal stability and enzymatic protection in SIF supplemented with pancreatin. The prototypes had high AE of 80–97%. To our knowledge, this is the first time that HA/CS PECs have been loaded with insulin in the absence of a cross-linker. The uncoated PECs demonstrated substantial insulin release in FaSSIF-V2 over 4 h and they were non-cytotoxic in vitro. At a loading of 500 µg/mL, EL100-coated PECs released insulin in a biphasic manner, which suggested that insulin was at least partly incorporated in the PEC coating. The instillation of coated PECs with the intestinal permeation enhancer, SL, to intact rat jejunal loops confirmed the bioactivity of entrapped insulin. While coated PECs had minimal effect following instillations, the co-administration with SL conferred efficacy and bioavailability.

## Figures and Tables

**Table 1:** Characterization of PECs that were loaded with a theoretical concentration of either 100 or 500 µg/mL insulin.

PEC	Loading (µg/mL)	Diameter (nm)	PdI	ZP (mV)	AE (%)	PL (%)	FL (µg/mg)
Uncoated	100	232 ± 36	0.15 ± 0.06	−33 ± 5	82 ± 6	8 ± 1	83 ± 7
	500	295 ± 58	0.28 ± 0.13	−36 ± 3	80 ± 2	31 ± 1	285 ± 13
Coated	100	154 ± 27	0.16 ± 0.07	−26 ± 8	95	1.5	17
	500	116 ± 28	0.21 ± 0.04	−23 ± 3	97 ± 1	6 ± 0	58 ± 5

AE (Association Efficiency), PL (Peptide Loading) and FL (Final Loading). Data expressed as mean ± SD (n = 3–7) except for EL100-coated PECs loaded with 100 µg/mL insulin (n = 2).

**Table 2:** Particle diameter, PdI and ZP of PECs used for in vivo studies before and after centrifugation, as measured by dynamic light scattering (DLS). The numbers in brackets are SD values.

PEC	Batches	Particle Diameter (nm)		PdI		ZP (mV)		C_f_
		Before	After	Before	After	Before	After	
Unloaded	1	175	298	0.16	0.16	−48	−53	6.71
500 µg/mL insulin, uncoated	4	200 (± 24)	307 (± 74)	0.23 (± 0.04)	0.25 (± 0.05)	−49 (± 1)	−54 (± 2)	3.17 (± 1.48)
500 µg/mL insulin, coated	2	87	105	0.15	0.24	−37	−59	4.05

Cf, Concentration Factor.

**Table 3:** Serum insulin pharmacokinetic data following jejunal instillation of 50 IU/kg insulin in PECs. Relative bioavailability (%F) was calculated relative to s.c. injection of insulin (1 IU/kg) over 120 min SL (100 mM). Instillation data unless stated otherwise. Data is expressed as mean ± SEM (n = 3–6 per group).

Treatment	AUC (_0–120 min_)	T_max_ (min)	C_max_ (mU/L)	%F(_0–120min_)
Insulin (s.c.)	6936 ± 2436	20 ± 0	111 ± 26	-
Coated PECs	1725 ± 411	30 ± 10	45 ± 14	0.5
Uncoated PECs + SL	3301 ± 784	23 ± 3	66 ± 16	1.0
Coated PECs + SL	4001 ± 2031	23 ± 3	142 ± 39	1.2

## References

[1] Hu Q., Luo Y. (2018). Recent advances of polysaccharide-based nanoparticles for oral insulin delivery. Int. J. Biol. Macromol..

[2] Batista P., Castro P.M., Madureira A.R., Sarmento B., Pintado M. (2018). Recent insights in the use of nanocarriers for the oral delivery of bioactive proteins and peptides. Peptides.

[3] Sonia T.A., Sharma C.P. (2012). An overview of natural polymers for oral insulin delivery. Drug Discov. Today.

[4] Nur M., Vasiljevic T. (2017). Can natural polymers assist in delivering insulin orally?. Int. J. Biol. Macromol..

[5] Wong C.Y., Al-Salami H., Dass C.R. (2017). Potential of insulin nanoparticle formulations for oral delivery and diabetes treatment. J. Control. Release.

[6] Hartig S.M., Greene R.R., Dikov M.M., Prokop A., Davidson J.M. (2007). Multifunctional nanoparticulate polyelectrolyte complexes. Pharm. Res..

[7] Bourganis V., Karamanidou T., Kammona O., Kiparissides C. (2017). Polyelectrolyte complexes as prospective carriers for the oral delivery of protein therapeutics. Eur. J. Pharm. Biopharm..

[8] Fallacara A., Baldini E., Manfredini S., Vertuani S. (2018). Hyaluronic acid in the third millennium. Polymers.

[9] Han L., Zhao Y., Yin L., Li R., Liang Y., Huang H., Pan S., Wu C., Feng M. (2012). Insulin-loaded pH-sensitive hyaluronic acid nanoparticles enhance transcellular delivery. AAPS PharmSciTech.

[10] Contreras-Ruiz L., de la Fuente M., Párraga J.E., López-García A., Fernández I., Seijo B., Sánchez A., Calonge M., Diebold Y. (2011). Intracellular trafficking of hyaluronic acid-chitosan oligomer-based nanoparticles in cultured human ocular surface cells. Mol. Vis..

[11] Al Rubeaan K., Rafiullah M., Jayavanth S. (2016). Oral insulin delivery systems using chitosan-based formulation: A review. Expert Opin. Drug Deliv..

[12] Smith J., Wood E., Dornish M. (2004). Effect of chitosan on epithelial cell tight junctions. Pharm. Res..

[13] Vllasaliu D., Exposito-Harris R., Heras A., Casettari L., Garnett M., Illum L., Stolnik S. (2010). Tight junction modulation by chitosan nanoparticles: Comparison with chitosan solution. Int. J. Pharm..

[14] Bilati U., Allémann E., Doelker E. (2005). Strategic approaches for overcoming peptide and protein instability within biodegradable nano- and microparticles. Eur. J. Pharm. Biopharm..

[15] Cárdenas-Bailón F., Osorio-Revilla G., Gallardo-Velázquez T. (2015). Microencapsulation of insulin using a W/O/W double emulsion followed by complex coacervation to provide protection in the gastrointestinal tract. J. Microencapsul..

[16] Hartig S.M., Carlesso G., Davidson J.M., Prokop A. (2007). Development of improved nanoparticle polyelectrolyte complex physicochemistry by non-stoichiometric mixing of polyions with similar molecular weights. Biomacromolecules.

[17] Schatz C., Domard C., Viton C., Pichot C., Delair T. (2004). Versatile and efficient formation of colloids of biopolymer-based polyelectrolyte complexes. Biomacromolecules.

[18] Umerska A., Paluch K.J., Inkielewicz-Stępniak I., Santos-Martinez M.J., Corrigan O.I., Medina C., Tajber L. (2012). Exploring the assembly process and properties of novel crosslinker-free hyaluronate-based polyelectrolyte complex nanocarriers. Int. J. Pharm..

[19] Umerska A., Paluch K.J., Martinez M.J.S., Corrigan O.I., Medina C., Tajber L. (2014). Self-assembled hyaluronate/protamine polyelectrolyte nanoplexes: Synthesis, stability, biocompatibility and potential use as peptide carriers. J. Biomed. Nanotechnol..

[20] Umerska A., Paluch K.J., Santos-Martinez M.J., Medina C., Corrigan O.I., Tajber L. (2015). Chondroitin-based nanoplexes as peptide delivery systems—Investigations into the self-assembly process, solid-state and extended release characteristics. Eur. J. Pharm. Biopharm..

[21] Umerska A., Corrigan O.I., Tajber L. (2014). Intermolecular interactions between salmon calcitonin, hyaluronate, and chitosan and their impact on the process of formation and properties of peptide-loaded nanoparticles. Int. J. Pharm..

[22] Ryan S.M., McMorrow J., Umerska A., Patel H.B., Kornerup K.N., Tajber L., Murphy E.P., Perretti M., Corrigan O.I., Brayden D.J. (2013). An intra-articular salmon calcitonin-based nanocomplex reduces experimental inflammatory arthritis. J. Control. Release.

[23] Sarmento B., Ribeiro A.J., Veiga F., Sampaio P., Neufeld R.J., Ferreira D. (2007). Alginate/chitosan nanoparticles are effective for oral insulin delivery. Pharm. Res..

[24] Lopes M., Aniceto D., Abrantes M., Simões S., Branco F., Vitória I., Botelho M.F., Seiça R., Veiga F., Ribeiro A. (2017). In vivo biodistribution of antihyperglycemic biopolymer-based nanoparticles for the treatment of type 1 and type 2 diabetes. Eur. J. Pharm. Biopharm..

[25] Sung H.-W., Sonaje K., Liao Z.-X. (2012). pH-responsive nanoparticles shelled with chitosan for oral delivery of insulin: From mechanism to therapeutic applications. Acc. Chem. Res..

[26] Sonaje K., Chen Y.J., Chen H.L., Wey S.P., Juang J.H., Nguyen H.N., Hsu C.W., Lin K.J., Sung H.W. (2010). Enteric-coated capsules filled with freeze-dried chitosan/poly(gamma-glutamic acid) nanoparticles for oral insulin delivery. Biomaterials.

[27] Yin L., Ding J., He C., Cui L., Tang C., Yin C. (2009). Drug permeability and mucoadhesion properties of thiolated trimethyl chitosan nanoparticles in oral insulin delivery. Biomaterials.

[28] Umerska A., Tajber L. (2015). Polyelectrolyte complexes as nanoparticulate drug delivery systems. Eur. Pharm. Rev..

[29] Thünemann A.F., Müller M., Dautzenberg H., Joanny J.F., Löwen H. (2004). Polyelectrolyte complexes. Adv. Polym. Sci..

[30] Plapied L., Duhem N., des Rieux A., Préat V. (2011). Fate of polymeric nanocarriers for oral drug delivery. Curr. Opin. Colloid. Interface Sci..

[31] McCartney F., Rosa M., Brayden D.J. (2019). Evaluation of sucrose laurate as an intestinal permeation enhancer for macromolecules: Ex Vivo and In Vivo studies. Pharmaceutics.

[32] De la Fuente M., Seijo B., Alonso M.J. (2008). Novel hyaluronan-based nanocarriers for transmucosal delivery of macromolecules. Macromol. Biosci..

[33] Malloy A., Carr B. (2006). Nanoparticle tracking analysis - the halo system. Part. Part. Syst. Charact..

[34] Dash R.N., Habibuddin M., Sahoo A., Kothawade A., Sachin N., Chaudhari M.R., Mahadik K.R. (2013). Factorial Approach for the development of stability indicating HPLC assay of recombinant human insulin: Application to its stability study. Curr. Pharm. Anal..

[35] Jintapattanakit A., Junyaprasert V.B., Mao S., Sitterberg J., Bakowsky U., Kissel T. (2007). Peroral delivery of insulin using chitosan derivatives: A comparative study of polyelectrolyte nanocomplexes and nanoparticles. Int. J. Pharm..

[36] Vertzoni M., Pastelli E., Psachoulias D., Kalantzi L., Reppas C. (2007). Estimation of intragastric solubility of drugs: In what medium?. Pharm. Res..

[37] Jantratid E., Jannsen N., Reppas C., Dressman J.B. (2008). Dissolution media simulating conditions in the proximal human gastrointestinal tract: An update. Pharm. Res..

[38] Corrigan D.O., Healy A.M., Corrigan O.I. (2006). Preparation and release of salbutamol from chitosan and chitosan co-spray dried compacts and multiparticulates. Eur. J. Pharm. Biopharm..

[39] Hubatsch I., Ragnarsson E.G.E., Artursson P. (2007). Determination of drug permeability and prediction of drug absorption in Caco-2 monolayers. Nat. Protoc..

[40] Cory A.H., Owen T.C., Barltrop J.A., Cory J.G. (1991). Use of an aqueous soluble tetrazolium/formazan assay for cell growth assays in culture. Cancer Commun..

[41] Morciano G., Sarti A.C., Marchi S., Missiroli S., Falzoni S., Raffaghello L., Pistoia V., Giorgi C., Di Virgilio F., Pinton P. (2017). Use of luciferase probes to measure ATP in living cells and animals. Nat. Protoc..

[42] Repetto G., del Peso A., Zurita J.L. (2008). Neutral red uptake assay for the estimation of cell viability/cytotoxicity. Nat. Protoc..

[43] Inkielewicz-Stepniak I., Tajber L., Behan G., Zhang H., Radomski M.W., Medina C., Santos-Martinez M.J. (2018). The role of mucin in the toxicological impact of polystyrene nanoparticles. Materials.

[44] Presas E., McCartney F., Sultan E., Hunger C., Nellen S., Alvarez C.V., Werner U., Bazile D., Brayden D.J., O’Driscoll C.M. (2018). Physicochemical, pharmacokinetic and pharmacodynamic analyses of amphiphilic cyclodextrin-based nanoparticles designed to enhance intestinal delivery of insulin. J. Control. Release.

[45] Nielsen E.J.B., Yoshida S., Kamei N., Iwamae R., Khafagy E.S., Olsen J., Rahbek U.L., Pedersen B.L., Takayama K., Takeda-Morishita M. (2014). In Vivo proof of concept of oral insulin delivery based on a co-administration strategy with the cell-penetrating peptide penetratin. J. Control. Release.

[46] Zhang Y., Huo M., Zhou J., Xie S. (2010). PKSolver: An add-in program for pharmacokinetic and pharmacodynamic data analysis in Microsoft Excel. Comput. Methods Programs Biomed..

[47] Wong C.Y., Luna G., Martinez J., Al-Salami H., Dass C.R. (2019). Bio-nanotechnological advancement of orally administered insulin nanoparticles: Comprehensive review of experimental design for physicochemical characterization. Int. J. Pharm..

[48] Swenson E.S., Milisen W.B., Curatolo W. (1994). Intestinal permeability enhancement: Efficacy, acute local toxicity, and reversibility. Pharm. Res..

[49] Garcia-Fuentes M., Prego C., Torres D., Alonso M.J. (2005). A comparative study of the potential of solid triglyceride nanostructures coated with chitosan or poly(ethylene glycol) as carriers for oral calcitonin delivery. Eur. J. Pharm. Sci..

[50] Sladek S., Kearney C., Crean D., Brama P.A.J., Tajber L., Fawcett K., Labberte M.C., Leggett B., Brayden D.J. (2018). Intra-articular delivery of a nanocomplex comprising salmon calcitonin, hyaluronic acid, and chitosan using an equine model of joint inflammation. Drug Deliv. Transl. Res..

[51] Dul M., Paluch K.J., Kelly H., Healy A.M., Sasse A., Tajber L. (2015). Self-assembled carrageenan/protamine polyelectrolyte nanoplexes—Investigation of critical parameters governing their formation and characteristics. Carbohydr. Polym..

[52] De la Fuente M., Seijo B., Alonso M.J. (2008). Novel hyaluronic acid-chitosan nanoparticles for ocular gene therapy. Investig. Ophthalmol. Vis. Sci..

[53] Boddohi S., Moore N., Johnson P.A., Kipper M.J. (2009). Polysaccharide-based polyelectrolyte complex nanoparticles from chitosan, heparin, and hyaluronan. Biomacromolecules.

[54] Sarmento B., Ribeiro A., Veiga F., Ferreira D. (2006). Development and characterization of new insulin containing polysaccharide nanoparticles. Colloids Surf. B Biointerfaces.

[55] Niu Z., Tedesco E., Benetti F., Mabondzo A., Montagner I.M., Marigo I., Gonzalez-Touceda D., Tovar S., Diéguez C., Santander-Ortega M.J. (2017). Rational design of polyarginine nanocapsules intended to help peptides overcoming intestinal barriers. J. Control. Release.

[56] Strand S.P., Tømmeraas T., Vårum K.M., Østgaard K. (2001). Electrophoretic light scattering studies of chitosans with different degrees of N-acetylation. Biomacromolecules.

[57] Cleland R.L., Wang J.L., Detweiler D.M. (1982). Polyelectrolyte properties of sodium hyaluronate. 2. Potentiometric titration of hyaluronic acid. Macromolecules.

[58] Chen R.H., Tsaih M.L., Lin W.C. (1996). Effects of chain flexibility of chitosan molecules on the preparation, physical, and release characteristics of the prepared capsule. Carbohydr. Polym..

[59] Cegnar M., Kerc J. (2010). Self-assembled polyelectrolyte nanocomplexes of alginate, chitosan and ovalbumin. Acta Chim. Slov..

[60] Voorhees P.W. (2005). The theory of Ostwald ripening. J. Stat. Phys..

[61] Ensign L.M., Cone R., Hanes J. (2012). Oral drug delivery with polymeric nanoparticles: The gastrointestinal mucus barriers. Adv. Drug Deliv. Rev..

[62] Chern C.S., Lee C.K., Chang C.J. (2004). Electrostatic interactions between amphoteric latex particles and proteins. Colloid Polym. Sci..

[63] Bal S.M., Slütter B., van Riet E., Kruithof A.C., Ding Z., Kersten G.F.A., Jiskoot W., Bouwstra J.A. (2010). Efficient induction of immune responses through intradermal vaccination with N-trimethyl chitosan containing antigen formulations. J. Control. Release.

[64] Jonassen H., Kjoniksen A.L., Hiorth M. (2012). Stability of chitosan nanoparticles cross-linked with tripolyphosphate. Biomacromolecules.

[65] Fernandes M., Gonçalves I.C., Nardecchia S., Amaral I.F., Barbosa M.A., Martins M.C.L. (2013). Modulation of stability and mucoadhesive properties of chitosan microspheres for therapeutic gastric application. Int. J. Pharm..

[66] Santander-Ortega M.J., Jódar-Reyes A.B., Csaba N., Bastos-González D., Ortega-Vinuesa J.L. (2006). Colloidal stability of Pluronic F68-coated PLGA nanoparticles: A variety of stabilisation mechanisms. J. Colloid Interface Sci..

[67] Tobío M., Sánchez A., Vila A., Soriano I., Evora C., Vila-Jato J.L., Alonso M.J. (2000). The role of PEG on the stability in digestive fluids and in vivo fate of PEG-PLA nanoparticles following oral administration. Colloids Surf. B Biointerfaces.

[68] Harde H., Agrawal A.K., Jain S. (2015). Tetanus toxoids loaded glucomannosylated chitosan based nanohoming vaccine adjuvant with improved oral stability and immunostimulatory response. Pharm. Res..

[69] Verheul R.J., Slütter B., Bal S.M., Bouwstra J.A., Jiskoot W., Hennink W.E. (2011). Covalently stabilized trimethyl chitosan-hyaluronic acid nanoparticles for nasal and intradermal vaccination. J. Control. Release.

[70] Momenzadeh S., Sadeghi A., Vatandoust N., Salehi R. (2015). Evaluation of in vivo transfection efficiency of Eudragit-coated nanoparticles of chitosan-DNA: A pH-sensitive system prepared for oral DNA delivery. Iran. Red Crescent Med. J..

[71] Zhang Y., Du X., Zhang Y., Li G., Cai C., Xu J., Tang X. (2014). Thiolated Eudragit-based nanoparticles for oral insulin delivery: Preparation, characterization, and evaluation using intestinal epithelial cells In Vitro. Macromol. Biosci..

[72] Tiyaboonchai W., Limpeanchob N. (2007). Formulation and characterization of amphotericin B-chitosan-dextran sulfate nanoparticles. Int. J. Pharm..

[73] Lopes M., Shrestha N., Correia A., Shahbazi M.A., Sarmento B., Hirvonen J., Veiga F., Seiça R., Ribeiro A., Santos H.A. (2016). Dual chitosan/albumin-coated alginate/dextran sulfate nanoparticles for enhanced oral delivery of insulin. J. Control. Release.

[74] Yao F., Weiyuan J.K. (2010). Drug release kinetics and transport mechanisms of non- degradable and degradable polymeric delivery systems. Expert Opin. Drug Deliv..

[75] Moustafine R.I., Margulis E.B., Sibgatullina L.F., Kemenova V.A., Mooter A., Van Den G. (2008). Comparative evaluation of interpolyelectrolyte complexes of chitosan with Eudragit L100 and Eudragit L100-55 as potential carriers for oral controlled drug delivery. Eur. J. Pharm. Biopharm..

[76] Calcetti P., Salmaso S., Walker G., Bernkop-Schnürch A. (2004). Development and in vivo evaluation of an oral insulin-PEG delivery system. Eur. J. Pharm. Sci..

[77] Thompson C.J., Tetley L., Uchegbu I.F., Cheng W.P. (2009). The complexation between novel comb shaped amphiphilic polyallylamine and insulin—Towards oral insulin delivery. Int. J. Pharm..

[78] Braia M., Tubio G., Nerli B., Loh W., DRomanini D. (2012). Analysis of the interactions between Eudragit^®^ L100 and porcine pancreatic trypsin by calorimetric techniques. Int. J. Biol. Macromol..

[79] Hong R., Fischer N.O., Verma A., Goodman C.M., Emrick T., Rotello V.M. (2004). Control of protein structure and function through surface recognition by tailored nanoparticle scaffolds. J. Am. Chem. Soc..

[80] McCartney F. (2016). Use of Permeation Enhancers and Nanotechnology to Increase Intestinal Peptide Permeability. Ph.D. Thesis.

[81] Henze L.J., Koehl N.J., O’Shea P., Kostewicz E.S., Holm R., Griffin B.T. (2019). The pig as a preclinical model for predicting oral bioavailability and in vivo performance of pharmaceutical oral dosage forms: A PEARRL review. J. Pharm. Pharmacol..

